# Effect of Surface Treatment and Resin Cement on the Bond Strength of an Advanced Lithium Disilicate

**DOI:** 10.1055/s-0043-1776358

**Published:** 2024-01-10

**Authors:** Yuqing Lu, Thera Elisa Bierman, Amanda Maria de Oliveira Dal Piva, João Paulo Mendes Tribst, Albert J. Feilzer, Cornelis J. Kleverlaan

**Affiliations:** 1 Department of Dental Materials Science, Academic Center for Dentistry Amsterdam (ACTA), Universiteit van Amsterdam and Vrije Universiteit, Amsterdam, The Netherlands; 2 Department of Reconstructive Oral Care, Academic Center for Dentistry Amsterdam (ACTA), Universiteit van Amsterdam and Vrije Universiteit, Amsterdam, The Netherlands

**Keywords:** ceramics, lithium disilicate, resin cement, surface properties, dental, bonding, shear strength

## Abstract

**Objectives** The aim of the study was to evaluate the effect of surface treatment and resin cement on the bond strength of conventional and advanced lithium disilicates (ALDs).

**Materials and Methods** Ceramic slices (2 × 13 × 15 mm) of conventional lithium disilicate (LD) (IPS e.max CAD) and ALD (CEREC Tessera) were sectioned, polished, and divided into 16 groups ( *n* = 10) according to the factors: ceramic, surface treatment, and resin cement (Panavia V5 and Variolink Esthetic DC). Surface treatments consisted of hydrofluoric acid 4.9% etching for 20 seconds ( *Hf20* ) or 30 seconds ( *Hf30* ), self-etching ceramic primer ( *Se* ), and sandblasting ( *Sb* ). Then, a resin cement cylinder (Ø = 2.5 mm) was manufactured on each specimen's surface. The specimens were then submitted to a shear bond strength (SBS) test. Surface roughness was evaluated through a contact profilometer, and surface morphology was evaluated under scanning electron microscopy for qualitative analysis.

**Statistical Analysis** Two-way analysis of variance (ANOVA) was used to analyze the data of SBS and surface roughness. For bond strength, the effects of surface treatment, resin cement, and the interaction were analyzed for each ceramic. For roughness, analyzed factors include ceramic and surface treatment.

**Results** ANOVA revealed that ceramic ( *p* = 0.047) and surface treatment ( *p* < 0.001) factors affected the bond strength, while the cements performed similarly. Both materials showed adequate bond strength (ALD 19.1 ± 7.7 MPa; LD 17.1 ± 7.9 MPa). *Sb* protocol showed the lowest mean value (9.6 ± 2.9 MPa) compared with *Hf20* (22.0 ± 7.1 MPa), *Hf30* (21.7 ± 7.4 MPa), and *Se* (19.3 ± 6.0 MPa).

**Conclusion** For both ceramics, the highest performance was obtained after applying Se and Hf20 or Hf30. Therefore, longer etching time is unnecessary. Sb protocol must be avoided.

## Introduction

Dental glass ceramics when first introduced in the market, in the early 1970s, was initially used for anterior restorations due to their esthetics. 1 However, they gradually became popular for posterior restorations as their strength and toughness improved. Since then, the development of dental glass ceramics has continued, with the introduction of newer materials that have even higher strength, various tooth-color shades and translucencies, and improved processing techniques. Glass ceramics such as lithium disilicate (LD) are the most popular known material, when it comes to esthetics, biocompatibility, and adhesion, 2 being widely used in a variety of dental applications, including crowns, bridges, inlays, onlays, and veneers.

Among the LDs, the first introduced CAD product, IPS e.max CAD, stands out with its flexural strength ranging from 350 to 440 MPa 3 and suitable bond strength ranging from 3.8 to 45.5 MPa depending on the surface treatment, ceramic primer, and cement. 4 5 The advanced lithium disilicate (ALD; CEREC Tessera, Dentsply Sirona) was released with quartz particles and a higher volume percentage of glass matrix 6 compared with conventional LD. The manufacturer claims that this ceramic has tooth-like esthetics, high strength, and a fast processing time. 7 ALD blocks are supplied in the final color since they already went through a firing cycle. Therefore, a shortened final firing cycle saves clinical time to complete its crystallization. 8 A previous study found that ALD presented superior hardness and surface smoothness compared with other evaluated glass ceramics. 2 It was also reported that ALD has a higher resistance to wear and presents similar fracture behavior compared with LD. 9 Furthermore, during a color stability analysis, ALD showed stable and similar color and translucency after coffee thermocycling compared with LD and zirconia-reinforced lithium silicate. 10

In addition to the restorative material's mechanical properties, mainly the durability of the adhesive bond determines the success of a glass ceramic restoration. As a consequence, its durability is strongly dependent on the adhesion procedures and cementation quality. Resin cement is the gold standard in the cementation of glass ceramics, advocated due to its resistance to water sorption and because its strong adhesive bond increases the strength of the restoration. 11 Considering the restoration thickness and translucency, either light-cured, dual-cured, or chemical resin cement can be used. However, before cementation, the ceramic surface treatment plays an important role in the bond strength. Different ceramics require different surface treatments according to their microstructure. However, for clinicians, it would be easier to standardize and simplify the surface treatments for different ceramics. As an example, single-bottle ceramic primers are available for adhesive procedures, but are still limited in terms of indications for different ceramic materials. 12 13

As a newly developed material, information regarding suitable surface treatments for ALD are still scarce. According to the manufacturer, the ALD surface should be etched for 30 seconds with 5% hydrofluoric acid ( *Hf30* ); 10 seconds more than the conventional LD. However, it is unknown if 20 seconds, as a standard procedure for LDs, would be sufficient to achieve suitable bond strength values for ALD. In addition, different surface treatments have been investigated to improve the bond strength between LD and resin cement, such as hydrofluoric acid ( *Hf* ) etchings followed by the silane application, *Se* , and sandblasting ( *Sb* ). 13 Studies reported that *Se* showed less damaged surface but similar bond strength to 5% *Hf* etching. 14 *Sb* , commonly used for zirconia ceramics, has not been advocated as an alternative for conventional LD due to its unfavorable performance. 13 However, whether *Se* or *Sb* can also be an option for ALD is still unknown.

This study aimed to investigate the effect of four different surface treatments on the bond strength between two dual-curing resin cements for LD or ALD. The hypotheses of this study consisted that (1) LD and ALD would present acceptable bond strength, (2) different surface treatments would affect the bond strength between the ceramics to the evaluated resin cements, and (3) both cements would present similar bond strength.

## Materials and Methods

### Specimens' Preparation

The information of the used materials is listed in Table 1 . Blocks of two LD ceramics (IPS e.max CAD [LD] and CEREC Tessera [ALD]) were sectioned in slices (2 × 13 × 15 mm) using a saw machine (Isomet 1000, Precision Sectioning Saw, Buehler, Lake Bluff, Illinois, United States) with a diamond disc and constant water cooling (Series 15LC Diamond Blade, Buehler). The slices were polished with a subsequent series of silicon carbide papers (#600, #800, and #1,200 grit) in a polishing machine (Ecomet/Automet 250, Buehler). After polishing, the ceramic slices were disinfected and cleaned in an ultrasonic ethanol bath, air-dried, and afterward crystallized in a ceramic oven (Programat P100, Ivoclar), according to their respective firing cycles: LD (closing time: 6 minutes, temperature gradient 1: 60°C/min, holding temperature 1: 770°C, holding time 1: 10 seconds, holding gradient 2: 30°C/min, holding temperature 2: 850°C, holding time 2: 10 minutes, vacuum 1: 550 until 770°C, vacuum 2: 770 until 850°C, long-term cooling: 700°C/min, and standby temperature: 403°C); ALD (closing time: 2 minutes, temperature gradient: 55°C/min, holding temperature: 760°C, holding time: 2 seconds, vacuum: off, long-term cooling: 0°C/min, and standby temperature: 403°C). Then, the ceramic slices were divided into 16 experimental groups ( *n* = 10/group), according to the ceramic, surface treatment, and resin cement (P—Panavia V5, Kuraray; V—Variolink Esthetic DC, Ivoclar). The group's distribution is summarized in Table 2 .

**Table 1:** Used materials' brand names, type, composition, and manufacturer

Brand name	Material type	Chemical composition
IPS e.max CAD a	Lithium disilicate Batch number: Z034M8	SiO _2_ , 57.0–80.0%; Li _2_ O, 11.0–19.0%; K _2_ O, 0.0–13.0%; P _2_ O _5_ , 0.0–11.0%; other oxides
CEREC Tessera b	Advanced lithium disilicate Batch number: 16011535	Glass zirconia matrix lithium disilicate Virgilite (LiAlSiO _6_ )
Monobond Etch & Primer a	Self-etching primer Batch number: Y48165	TADF, silane methacrylate, BTSE, methacrylated phosphoric acid ester, butanol, water, colorant (pH = 3.7)
Monobond Plus a	Ceramic primer Batch number: Y45831	0-Methacryloyloxydecyl dihydrogen phosphate, methacrylated phosphoric acid ester, adhesive monomers, ethanol
Clearfill Ceramic Primer Plus c	Ceramic primer Batch number: 1C0071	3-Methacryloxypropyl trimethoxy silane, 10-methacryloyloxydecyl dihydrogen phosphate, ethanol
IPS Ceramic Etching Gel a	Hydrofluoric acid Batch number: Y48112	4.9% HF acid, water, colorant (pH = 2)
White Aluminum Oxide d	Aluminum oxide Batch number: L1MPW	50 µm Al _2_ O _3_
Panavia V5 a b c	Dual-curing resin cement Batch number: 1Q0188	A paste: Bis-GMA, TEGDMA, hydrophobic aromatic dimethacrylate, hydrophilic aliphatic dimethacrylate, silanated barium glass filler, fluoroaluminosilicate glass filler, colloidal silica, accelerator, initiator B paste: Bis-GMA, hydrophobic aromatic dimethacrylate, hydrophilic aliphatic dimethacrylate, silanated barium glass filler, silanated aluminum oxide filler, accelerator, dl-camphorquinone, pigments 39
Variolink Esthetic DC a	Dual-curing resin cement Batch number: Z02YY1	UMDA, methacrylate monomers as 1,10-decandiol dimethacrylate, a-dimethylbenzyl hydroperoxide, initiators, stabilizers, pigments and inorganic fillers of ytterbium trifluoride, spheroid mixed oxide (particle size: 0.04–0.2 μm. Mean particle size: 0.1 μm and 67 wt% = 38 vol%) 40

a Ivoclar, Liechtenstein.

b Dentisply Sirona, Germany.

c Kuraray Noritake Dental Inc., Japan.

d Danville Materials, Germany.

**Table 2:** Group's distribution, shear bond strength (MPa) mean ± standard deviation, and Tukey grouping a according to ceramic, surface treatment, and resin cement

Group	Ceramic	Surface treatment	Resin cement	Shear bond strength
LD *Hf20* P	LD	4.9% Hydrofluoric acid etching for 20 s + ceramic primer	Panavia V5	22.00 ± 5.04 ^A^
LD *Hf20* V			Variolink Esthetic DC	19.86 ± 9.31 ^A^
LD *Hf30* P		4.9% Hydrofluoric acid etching for 30 s + ceramic primer	Panavia V5	20.16 ± 6.98 ^A^
LD *Hf30* V			Variolink Esthetic DC	22.08 ± 7.71 ^A^
LD *Se* P		Self-etching ceramic primer	Panavia V5	19.28 ± 5.99 ^A^
LD *Se* V			Variolink Esthetic DC	17.15 ± 4.79 ^A^
LD *Sb* P		Sandblasting + ceramic primer	Panavia V5	8.83 ± 3.72 ^B^
LD *Sb* V			Variolink Esthetic DC	7.87 ± 2.69 ^B^
ALD *Hf20* P	ALD	4.9% Hydrofluoric acid etching for 20 s + ceramic primer	Panavia V5	23.49 ± 6.22 ^A^
ALD *Hf20* V			Variolink Esthetic DC	22.52 ± 7.54 ^A^
ALD *Hf30* P		4.9% Hydrofluoric acid etching for 30 s + ceramic primer	Panavia V5	21.38 ± 6.66 ^A^
ALD *Hf30* V			Variolink Esthetic DC	23.20 ± 8.74 ^A^
ALD *Se* P		Self-etching ceramic primer	Panavia V5	20.51 ± 5.48 ^A^
ALD *Se* V			Variolink Esthetic DC	20.14 ± 7.71 ^A^
ALD *Sb* P		Sandblasting + ceramic primer	Panavia V5	10.77 ± 1.98 ^B^
ALD *Sb* V			Variolink Esthetic DC	10.92 ± 1.97 ^B^

Abbreviations: ALD, advanced lithium disilicate; *Hf20* , hydrofluoric acid etching for 20 seconds; *Hf30* , hydrofluoric acid etching for 30 seconds; LD, lithium disilicate; *Se* , self-etching ceramic primer; *Sb* , sandblasting.

a Same capital letter means statistically similar bond strength between groups within similar ceramic material.

### Surface Treatments

For both evaluated ceramics, the surface treatments followed the procedures described below:

*Hf20* and *Hf30* : The surfaces were treated with 4.9% Hydrofluoric acid ( *Hf* ) for 20 or 30 seconds, respectively. Then, a layer of ceramic primer was applied and allowed to react for 60 seconds. After that, a water- and oil-free airstream was used to remove excess material.

Self-etching ceramic primer ( *Se* ): *Se* was actively applied using a microbrush for 20 seconds. Then, it was allowed to react for another 40 seconds. Then, the primer was washed off and dried for 10 seconds.

*Sb* : The surfaces were sandblasted (10 seconds, 10 mm, and 2.8 bar) with 50 µm aluminium oxide 13 using a *Sb* machine (ESPE Rocatector delta, Industrie Forum Design Hannover). Then, the surfaces were cleaned in an ultrasonic ethanol bath and air-dried before receiving a layer of ceramic primer as described earlier.

In sequence, polytetrafluoroethylene tubes with an internal diameter of 2.5 mm were used to build 10 cylinders per group with dual-curing resin cements (Panavia V5 or Variolink Esthetic DC, respectively, subgroups P and V). 15 16 Both cements were applied with a mixing tip and light cured for 10 seconds on both sides (from the top and the bottom, through the ceramic) using a light-curing lamp (1,200 mW/cm ^2^ , Elipar S10, 3M ESPE). Next, the tubes were removed directly after light curing while the cement cylinder remained on the ceramic. The specimens were stored for 72 hours in distilled water at a temperature of 37°C, to allow the complete polymerization curing of the resin cement before testing.

### Surface Roughness and Morphology

To investigate the effect of each surface treatment on the ceramics' surface, one representative specimen from each group was analyzed using a contact profilometer (SJ 400, Mitutoyo, Tokyo, Japan). Ten measurements were performed with a read length of 3 mm and speed of 0.2 mm/s, considering three different parameters: Ra (absolute average roughness of the heights of the irregularities along the profile), Rz (average maximum height of the profile), and RSm (spacing or average width of the profile of irregularities). The analysis followed ISO 4287-1997 standards, with a Gaussian filter and cutoff wavelength value of 0.8 mm. 17

Before and after surface treatments, representative specimens' surfaces were evaluated under scanning electron microscopy (SEM; EVO LS15, Zeiss, Germany) to investigate the surface morphology of each group. For that, all specimens received a gold coat in a low-pressure atmosphere.

### Shear Bond Strength Test

The specimens were vertically placed in a standardized setup with minimum space between the specimen and the holder to allow free movement and maintain a straight vertical position. The specimens were loaded till failure using a universal testing machine (Instron 6022; Instron Limited, High Wycombe, UK; loadcell = 1,000 N and crosshead speed = 0.5 mm/min). Then, the strength (in MPa) was calculated using the following formula:



where *F* is load at failure (in N) and *r* is the radius (in mm) of the interfacial area. All failed specimens were examined by two observers using a stereomicroscope (Stemi SV6, Zeiss, Germany). The failures were classified as: (1) adhesive failure, (2) mixed failure of adhesive and cohesive of cement or ceramic, (3) cohesive failure of ceramic, or (4) cohesive failure of cement.

### Statistical Analysis

The normality of the data was confirmed using the Ryan–Joiner's test. According to the literature data, the threshold of a shear bond strength (SBS) value between 10 and 13 MPa was considered as the minimum necessary between resin cement and ceramic. 18 19 Then, two-way analysis of variance (ANOVA) was used to analyze the SBS data of each ceramic considering the effects and the interaction of the factors: surface treatment and resin cement. For surface roughness, two-way ANOVA was applied to investigate the factors: ceramic material and surface treatment. The grouping distribution was analyzed using Tukey's test. For all tests, a confidence interval of 95% was chosen and *p* -values of less than 0.05 were considered statistically significant. The analyses were performed using a statistical software program (Minitab 18, Pennsylvania, United States).

## Results

### Surface Roughness

Factors ceramic, surface treatment, and their interaction influenced the average surface roughness Ra and Rz ( *p* < 0.001). RSm parameter was affected by surface treatment and its interaction with ceramic, respectively, *p* = 0.007 and *p* < 0.001. Surface roughness results are summarized in Table 3 .

**Table 3:** Mean and standard deviation of surface roughness parameters (Ra, Rz, and RSm in μm) and grouping a according to ceramic (LD and ALD) and surface treatment ( *Hf20* , *Hf30* , *Se* , and *Sb* )

Ceramics after surface treatment	Ra	Rz	RSm
LD *Hf20*	0.06 ± 0.00 ^C^	0.71 ± 0.10 ^C^	104.6 ± 50.1 ^BC^
LD *Hf30*	0.06 ± 0.01 ^C^	0.73 ± 0.25 ^C^	154.4 ± 102.1 ^BC^
LD *Se*	0.05 ± 0.00 ^C^	0.32 ± 0.04 ^C^	304.3 ± 215.4 ^A^
LD *Sb*	1.43 ± 0.10 ^B^	10.45 ± 0.71 ^B^	135.9 ± 17.8 ^BC^
ALD *Hf20*	0.04 ± 0.00 ^C^	0.42 ± 0.10 ^C^	98.0 ± 33.8 ^C^
ALD *Hf30*	0.05 ± 0.01 ^C^	0.59 ± 0.16 ^C^	159.7 ± 83.9 ^ABC^
ALD *Se*	0.03 ± 0.00 ^C^	0.33 ± 0.11 ^C^	121.9 ± 132.0 ^BC^
ALD *Sb*	2.50 ± 0.25 ^A^	17.17 ± 2.30 ^A^	245.6 ± 47.4 ^AB^

Abbreviations: ALD, advanced lithium disilicate; *Hf20* , hydrofluoric acid etching for 20 seconds; *Hf30* , hydrofluoric acid etching for 30 seconds; LD, lithium disilicate; *Se* , self-etching ceramic primer; *Sb* , sandblasting.

a Same capital letter means statistically similar roughness between groups considering ceramic and surface treatment.

For the ceramic material, ALD showed higher mean values (Ra [0.65 ± 1.09 μm] ^A^ and Rz [4.63 ± 7.42 μm] ^A^ ) than LD (Ra [0.40 ± 0.61 μm] ^B^ and Rz [3.05 ± 4.34 μm] ^B^ ). For surface treatment, ANOVA revealed that *Sb* promoted higher Ra (1.97 ± 0.58 μm) ^A^ and Rz (13.81 ± 3.83 μm) ^A^ than the other treatment groups: *Hf20* : Ra (0.05 ± 0.01 μm) ^B^ and Rz (0.57 ± 0.18 μm) ^B^ , *Hf30* : Ra (0.05 ± 0.01 μm) ^B^ and Rz (0.66 ± 0.22 μm) ^B^ , and *Se* : Ra (0.04 ± 0.01 μm) ^B^ and Rz (0.33 ± 0.08 μm) ^B^ . Parameter RSm showed that *Se* (213.1 ± 197.4 μm) ^A^ , *Sb* (190.7 ± 66.2 μm) ^A^ , and *Hf30* (157.0 ± 91.0 μm) ^AB^ present similar and higher spacing between defects compared with *Hf20* (101.3 ± 41.7 μm) ^B^ .

Considering the interaction of ceramic and surface treatment, ALD *Sb* (Ra: [2.50 ± 0.25 μm] ^A^ , Rz: [17.17 ± 2.30 μm] ^A^ ) promoted the highest Ra and Rz values followed by LD *Sb* (Ra: [1.43 ± 0.10 μm] ^B^ , Rz: [10.45 ± 0.71 μm] ^B^ ), while there was no significant difference between all the other groups. For RSm, LD *Se* (304.3 ± 215.4 μm) ^A^ promoted the highest mean spacing between defects for all the experimental groups, but it showed no significant difference compared with ALD *Sb* (245.6 ± 47.4 μm) ^AB^ and ALD *Hf30* (159.7 ± 83.9 μm) ^ABC^ ; ALD *Hf20* (98.0 ± 33.8) ^C^ generated the lowest mean spacing, which was similar to LD *Hf20* (104.6 ± 50.1 μm) ^BC^ , ALD *Se* (121.9 ± 132.0 μm) ^BC^ , LD *Sb* (135.9 ± 17.8 μm) ^BC^ , LD *Hf30* (154.4 ± 102.1 μm) ^BC^ , and ALD *Hf30* (159.7 ± 83.9 μm) ^ABC^ .

**Fig. 1:**
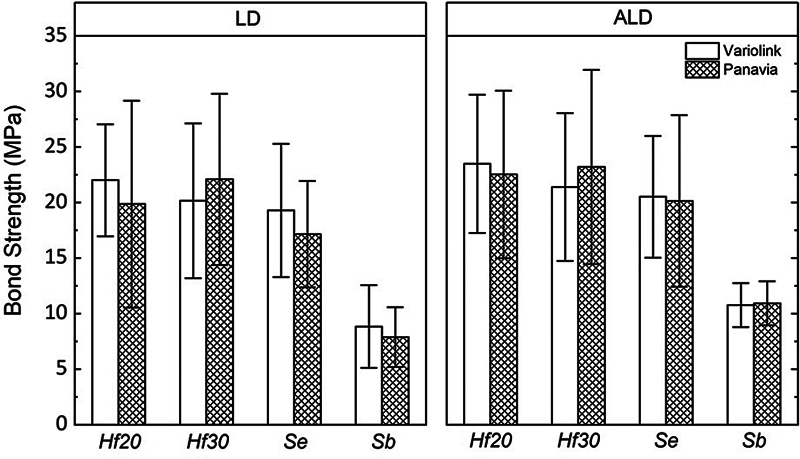
Bar chart of bond strength according to the evaluated ceramics (advanced lithium disilicate [ALD] and lithium disilicate [LD]), resin cements, and surface treatment: hydrofluoric acid etching for 20 seconds ( *Hf20* ) or 30 seconds ( *Hf30* ), self-etching ceramic primer ( *Se* ), and sandblasting ( *Sb* ).

SEM images showed qualitative surface morphology changes in the *Sb* compared with the other protocols. Moreover, in the *Sb* and control groups (Ctr, after polishing), ALD surfaces were rougher than LD ( Fig. 1 ).

### Shear Bond Strength

**Fig. 2:**
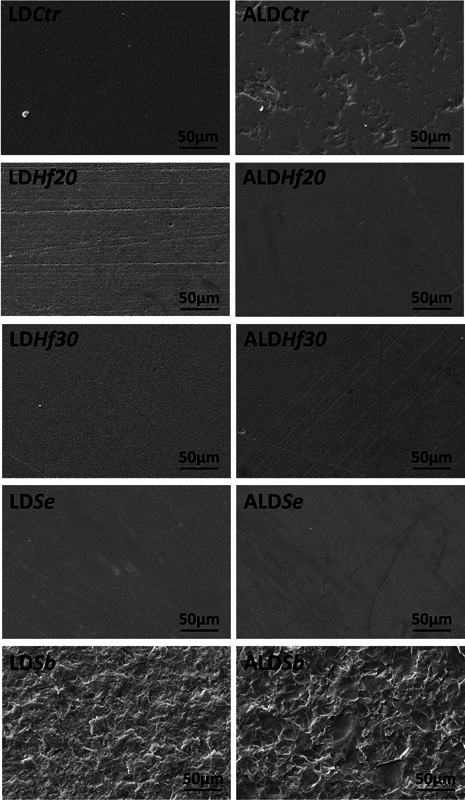
SEM images (×1,000) according to the ceramic (lithium disilicate [LD] and advanced lithium disilicate [ALD]) and surface treatment (control [ *Ctr* , after polishing], hydrofluoric acid etching for 20 seconds [ *Hf20* ], hydrofluoric acid etching for 30 seconds [ *Hf30* ], self-etching primer [ *Se* ], and sandblasting [ *Sb* ]).

Two-way ANOVA for LD and ALD revealed that the SBS was affected only by surface treatment ( *p* < 0.001), without significant difference between the evaluated cements ( *p* > 0.05) and for the interaction of factors ( *p* > 0.05). The mean and standard deviation are presented in Table 2 and Fig. 2 . Both ALD and conventional LD showed acceptable bond strength values (19.1 ± 7.7 and 17.1 ± 7.9 MPa, respectively). For ALD, *Hf20* (23.0 ± 6.8 MPa) ^A^ , *Hf30* (22.3 ± 7.6 MPa) ^A^ , and *Se* (20.3 ± 6.5 MPa) ^A^ promoted higher bond strength compared with *Sb* (10.8 ± 1.9 MPa) ^B^ , while for LD, *Hf30* (21.1 ± 7.2 MPa) ^A^ , *Hf20* (20.9 ± 7.4 MPa) ^A^ , and *Se* (18.2 ± 5.4 MPa) ^A^ promoted higher bond strength compared with *Sb* (8.4 ± 3.2 MPa) ^B^ .

According to the failure analysis, the most prevalent failure types were adhesive ( *n* = 80; 50%) and mixed failures of the adhesive and cohesive of cement or ceramic ( *n* = 80; 50%). There were no complete cohesive failures. Considering the mixed failures, 14% ( *n* = 11) presented cohesive failure of the ALD.

## Discussion

The purpose of this study was to evaluate the effect of different surface treatments on the bond strength between two resin cements and two different LDs: LD and ALD. A previous study reported that ALD can reach a similar bond strength as LD by applying the respective manufacturer-recommended protocol. 20 The results of this study showed that both ALD and LD showed acceptable bond strength values, accepting the first hypothesis. Although both materials showed comparable bond strength data, there is a trend of high bond strength averages in ALD. The present results can be justified because ALD contains fewer silicate crystals and more glass matrix compared with LD. 6 Therefore, during the etching procedure, more glass matrix is dissolved which creates a surface with improved micromechanical features and, therefore, adequate bond strength to the resin cements. 6 21

The evaluated surface treatments affected the bond strength for both ceramics, accepting the hypothesis that different surface treatments would affect the bond strength between LD and ALD regardless of the used resin cement. Results show that *Hf* etching followed by a ceramic primer application or a *Se* application promoted the highest mean bond strength values. Equivalent bond strength values with *Hf* and *Se* have already been reported for LD. 13 14 Therefore, this study reinforces that not only for LD but also for ALD, the same bonding procedure can be used. The manufacturer from ALD indicates *Hf30* treatment; however, no difference was observed between etching time for 20 or 30 seconds or *Se* . The results corroborate with previous studies that evaluated the bond strength of LD and determined that an etching time longer than 20 seconds for *Hf* etching does not improve its bond strength. 22 23

Regarding the evaluated resin cement, no difference has been observed in terms of SBS. Variolink Esthetic DC and Panavia V5 have both proven themselves with high bond strengths to LD, respectively, with 32.5 and 22.5 MPa. 11 21 Both are dual-curing resin cement indicated to cement glass ceramic restorations such as LD and ALD. According to the literature, while for enamel and dentin, 17 to 20 MPa are needed to resist stresses from the resin cement polymerization shrinking 24 ; an SBS value between 10 and 13 MPa is the minimum necessary between resin cement and ceramics. 18 19 The main purpose of evaluating two different types of cement was to reinforce or not the values obtained for one product. In addition, this analysis will allow data comparison between previous studies with LD and future studies with ALD. Results corroborate with the literature regarding the observed SBS between LD and resin cement, with mean values of approximately 17 MPa. 25 26 27

This study was the first to compare the effects of different surface treatments on the bond strength of ALD with different types of resin cement and presents a clinical recommendation that differs from the current manufacturer's instructions. Therefore, results offer original findings that allow dental clinic practitioners to improve their work. Since it justifies similar bonding procedure's possibilities for LD and ALD, it reduces the chance of errors in this initial stage of the cementing process, allowing the use of *Hf* with reduced exposition time. Additionally, it also allows the possibility to reduce the number of clinical steps for ALD when using the *Se* protocol.

In this study, surface roughness was investigated to examine its effect on the SBS. To determine the difference between similar glass materials, this study considered three different surface roughness parameters: Ra, Rz, and RSm. According to the findings, the surface treatments affected the surface roughness of both ceramics. *Hf20* , *Hf30* , and *Se* presented similar Ra and Rz, and higher SBS compared with *Sb* which had the highest surface roughness. Micromechanical retention benefits the bond strength and durability. However, surface roughness above certain microlevels can result in dampening of the micromechanical retention. 28 SEM images corroborate that *Sb* roughened LD and ALD surfaces and caused surface damage, which decreased the bond strength. This can be justified because *Sb* with alumina particles results in abrasion of the glass matrix and LD crystals, resulting in a weakened surface with lower bond strength values. 28 According to a meta-analysis, 29 roughness (Ra parameter) does not increase significantly in LD using 4.9 to 5% *Hf* for 20 seconds compared with the polished control. In addition, Ra values corroborated with previously published mean values in the literature. 30 31

Adhesive failure was the most prevalent failure type, followed by mixed failures resulting from an adhesive bond strength higher than the cohesive strength of cement or ceramic. 32 The presence of ceramic cohesive failure in the mixed failures was only observed for ALD. It can be explained that although ALD had higher bond strength benefiting from its higher amount of glass matrix, fewer silicate crystals could weaken its intrinsic strength. 33 Thus, its bond strength could be sometimes even stronger than the ceramic strength, resulting in the cohesive failure of ALD.

As a limitation of this study, it evaluated the effect of different surface treatments on the immediate bond strength between LD and ALD to different types of resin cement. Therefore, further studies considering aging protocols are strongly advocated to investigate the bond strength in long-term behavior. 34 35 36 In addition, as an *in vitro* study, this research did not consider all the factors present in the oral medium, such as fatigue 37 and pH variation. Clinical studies are also suggested to corroborate or contradict the present findings. Additionally, the study exclusively focused on bond strength as the primary outcome measure. Future investigations could encompass additional aspects such as marginal adaptation, color stability, and overall restoration longevity, providing a more comprehensive understanding of the material interactions and clinical implications. 37 38

Within the limitations of the current study, it can be concluded that ALD and LD showed acceptable bond strength; *Hf* etching and *Se* promote high SBS values for both evaluated ceramics; similar bond strength was found for both evaluated resin cements; *Sb* promoted higher surface roughness but lower bond strength compared with *Hf* etching and *Se* . In addition, the appropriately shortened time of *Hf* etching does not affect bonding performance. Conversely, the excessive etching time could increase the health risk for dental practitioners due to the toxicity of *Hf* . Despite longer operation time, the *Se* offers a safer alternative from this perspective.

## Conclusion

*Hf20* and *Se* are suitable surface treatments for both LD and ALD. *Hf30* did not improve bond strength. *Sb* significantly weakens the bond strength, and it is therefore not recommended.
